# Regulatory T Cell Ablation Causes Acute T Cell Lymphopenia

**DOI:** 10.1371/journal.pone.0086762

**Published:** 2014-01-23

**Authors:** Bruno Moltedo, Saskia Hemmers, Alexander Y. Rudensky

**Affiliations:** 1 Howard Hughes Medical Institute, Memorial Sloan Kettering Cancer Center, New York, New York, United States of America; 2 Immunology Program, Memorial Sloan Kettering Cancer Center, New York, New York, United States of America; Keio University School of Medicine, Japan

## Abstract

Regulatory T (Treg) cells enforce T cell homeostasis and maintain peripheral T cell tolerance. Here we report a previously unappreciated phenomenon of acute T cell lymphopenia in secondary lymphoid organs and non-lymphoid tissues triggered by Treg cell depletion that precedes the expansion of self-reactive T cells. Lymphopenia affects both neonates and adults indicating a dominant role of Treg cells in maintaining peripheral T cell numbers regardless of the developmental stage. The lymphopenia was neither triggered by caspase-dependent apoptosis nor macrophage-mediated clearance of T cells, nor diminished survival of naïve or recently activated T cells due to paucity of IL-7. It is possible that transient lymphopenia associated with congenital or acute Treg cell deficiency may contribute to the development of T cell mediated autoimmune disorders.

## Introduction

T cell tolerance is established early during thymic T cell development and later is enforced in the periphery in tissues and secondary lymphoid organs. The production of a diverse T cell receptor (TCR) repertoire in the thymus is thought to favor effective adaptive immune responses to pathogens but comes at the price of generating self-reactive T cells [1]. Although thymic negative selection is thought to eliminate almost all T cells bearing TCR specificities with strong affinity for self peptide-MHC complexes (pMHC) [2], [3], the mature T cell pool harbors self-reactive T cells capable of causing severe autoimmune disorders unless additional tolerance mechanisms operate properly in the periphery [4], [5].

Peripheral T cell tolerance in neonates as well as in adults is dependent on a specialized subset of CD4^+^ T cells known as regulatory T (Treg) cells, whose differentiation and function is dependent upon expression of the X-chromosome encoded transcription factor Foxp3 [6]. Humans with *Foxp3* loss-of-function mutations succumb to a CD4^+^ T cell mediated autoimmune disorder known as IPEX (Immune dysregulation, polyendocrinopathy, enteropathy, X-linked) syndrome [7], [8], [9]. In mice, Treg cell deficiency due to a spontaneous frame-shift mutation in the *Foxp3* gene (*scurfy*) or its targeted deletion results in a fatal neonatal autoimmune disorder, which can be prevented by adoptive transfer of Treg cells [10], [11]. Furthermore, Treg cell ablation in adult mice triggers a fatal autoimmune disease demonstrating the critical role of these cells in sustaining peripheral T cell homeostasis throughout the lifetime of the host [12].

A major role for T cell lymphopenia has been implicated in breaking peripheral T cell tolerance in both young and adult mice [13], [14]. Transient acute lymphopenia can be induced experimentally by the administration of drugs such as cyclophosphamide and cyclosporine [15], [16] or repetitive low dose irradiation [17], [18]. However, the additional disruption of thymic function either by thymectomy or by exposure to ionizing radiation is required for autoimmunity to commence in these models [15], [18]. Thymectomy exacerbates lymphopenia due to impaired thymic output of new T cells and compromised thymic Treg cell development. The lymphopenic environment and Treg cell deficiency also predispose to autoimmune disease commencing upon transfer of naïve T cells into T cell-deficient mice [6], [19]. On the other hand, experimental ablation of Treg cells seemingly causes autoimmunity in the absence of lymphopenia. Indeed, both, adult lymphoreplete and neonatal lymphopenic *Foxp3^DTR^* mice develop fatal T cell mediated autoimmune disease upon sustained Treg cell depletion [12], [20]. Here, we report a previously unappreciated transient lymphopenia following Treg cell ablation *in vivo* in neonate and adult mice. This early event was restricted to the peripheral, but not thymic T cell pool and preceded self-reactive T cell proliferation and activation *in vivo*.

## Materials and Methods

### Ethics Statement

All animals were handled in strict accordance with good animal practice as defined by relevant national and institutional guidelines. Animal work was approved by the Memorial Sloan-Kettering Cancer Center Institutional Animal Care and Use Committee (IACUC) (protocol # 08-10-023).

### Mice

*Foxp3^WT^* and Ly5.1 and Ly5.2 *Foxp3^DTR^* mice [12] on a B6 background (backcrossed >16 generations) were housed and bred in the specific pathogen–free facility at the Memorial Sloan-Kettering Cancer Center. For studies of Treg cell depletion in adults, mice were used at 6–8 weeks of age. For studies of Treg cell depletion in neonates, *Foxp3^DTR^* female mice were mated overnight with male breeders, and checked for vaginal plugs in the mornings. Plugged females were considered E-0.5 and separated from male mice. Female mice were monitored throughout the pregnancy and the time of birth was considered day 0. Neonates were used for experiments on day 3 after birth.

### *In vivo* Treg Cell Ablation

Treg cell ablation in adult *Foxp3^DTR^* mice was accomplished by a single i.p injection of DT (Sigma) at a dose of 50 mg/kg. Mice were analyzed at day 2 after DT treatment (DTx). Day 3 neonates were injected i.p using a Hamilton syringe, needle size 33G, with 100 ng DT in 20 µL PBS, and mice were analyzed at day 3 and 4 after DTx.

### Neonatal Thymic Output

Neonates were anesthesized by hypothermia. Thymocytes were labeled *in situ* by injecting each thymus with 20 µL of 10 mg/mL Alexa Fluor-647 succinimidyl ester in PBS. After labeling, mice were injected i.p with DT or PBS. Ratios of Alexa-647^+^ CD4^+^ T cells in the thymus and spleen in individual mice were determined on day 4 after DTx.

### Adoptive Transfers and Flow Cytometry

Spleen, skin draining axillary and inguinal lymph nodes, lung and liver cells were isolated by mechanical dissociation followed by digestion with collagenase-D (1 mg/mL; Roche) for 30 min at 37°C in DMEM, 1% FCS, 1.2 mM CaCl_2_. Cell numbers were measured using a Guava analyzer (Guava Technologies). Cell suspensions were filtered through 70 µm strainers and stained for flow cytometric analysis with the following monoclonal antibodies: anti-CD45.2 (104), anti-CD45.1 (A20), anti-TCRβ (H57-597), anti-CD4(GK1.5, RM4-5), anti-CD8α GK1.5, RM4-5), anti-Foxp3 (FJK-16s), anti-CD62L (MEL-14), anti-CD44 (IM7), anti-CD69 (H1.2F3), anti-CD25(PC61), anti-CD11c(N418), anti-MHC-II (M5/114.15.2), anti-CD11b(M1/70), anti-Gr1(RB6-8C5), anti-CD115(AFS98), anti-CD49b(DX5), anti-NK1.1(PK136), anti-B220(RA3-6B2), anti-CD19(6D5), anti-F4/80(F4/80), anti-Ki67 (Ki-67), anti-IFNγ (XMG1.2,R4-6A2). Antibodies were purchased from eBioscience, BD Biosciences or Biolegend. Apoptosis was measured by incubating the indicated single cell suspensions with Annexin-V (BD Bioscience) according to manufacturer’s instructions. Cell samples were acquired using an LSR-II (BD Biosciences) flow cytometer and analyzed with FlowJo software (Treestar).

Ly5.1^+^ or Ly5.1^+^ Ly5.2^+^ T cells from *Foxp3^DTR^* or *Foxp3^WT^* were purified by negative sorting on magnetic beads according to manufacturer instructions (Miltenyi). T cells were labeled with CellTrace Violet (2.5 µM; Invitrogen), and 1×10^6^ cells of each population were transferred i.v. to recipient Ly5.2 *Foxp3^DTR^* mice 1 day prior to DT injection.

### Inhibition of Apoptosis *in vivo*

Foxp3*^DTR^* mice were injected i.p with the cell-permeable pan-caspase inhibitor QVD-OPH (SM Biochemicals LLC) at 20 mg/kg body weight on the day of, and one day after DTx. 2 days after DTx, the indicated cell populations were isolated from the mice and analyzed by flow cytometry.

### IL-7 Treatment and Macrophage Depletion

*Foxp3^DTR^* mice were administered with recombinant human IL-7 (rh-IL7) at 0, 12, 24, and 36 h post DT injection (0.5 µg i.v. each). Splenic macrophages were depleted in *Foxp3^DTR^* mice by an i.v. injection of clodronate liposomes (100 µL) 10 days prior to Treg cell ablation. Cell preparations were isolated from mice and analyzed at day 2 after DTx.

### Quantitative RT-PCR and ELISA

Total RNA was isolated using Trizol (Invitrogen) from skin draining lymph nodes and spleens of Foxp3*^DTR^* mice injected with DT or PBS at different times after DTx. Samples were homogenized in 3 mL of Trizol reagent and mRNA was isolated according to manufacturer’s instructions. RT-PCR was performed using the Superscript III First Strand Kit (Invitrogen). Gene expression levels were determined by qPCR based on SyBR Green detection (Power SYBR Green PCR Master Mix, Applied Biosystems). Reactions were performed in 384 well plates using a 7900HT Fast Real-Time PCR System (Applied Biosystems). Gene expression levels were normalized to α-tubulin mRNA.

### Statistical Analysis

Data was analyzed using Prism software (Graphad, CA). Statistical analysis was performed via an unpaired Student’s *t* test at a 95% confidence interval. *p* values below 0.05 were considered significant: p<0.05 (*), p<0.01(**), p<0.001(***), p<0.0001(****).

## Results and Discussion

### Treg Ablation Results in a Marked Decrease in T Cell Numbers in Neonates

**Figure 1:**
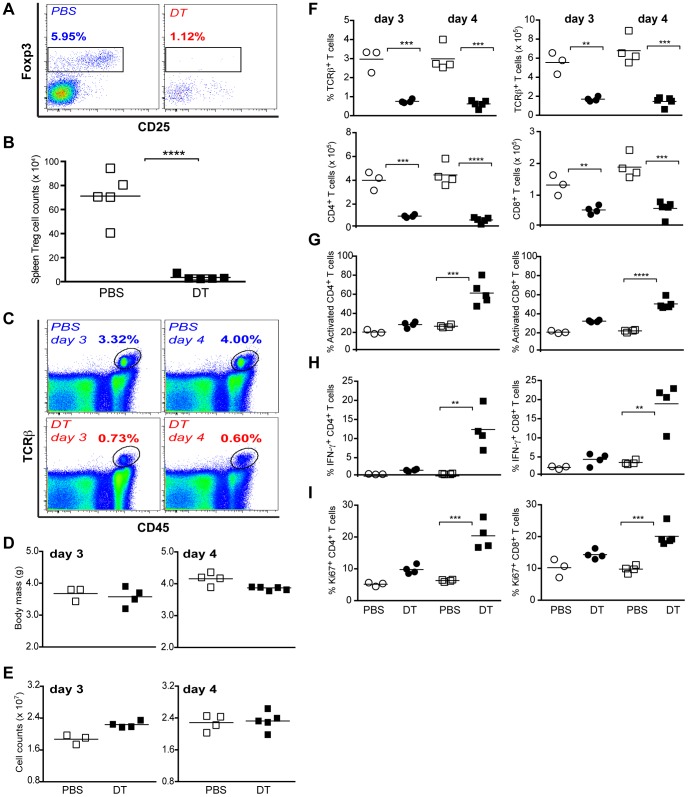
Treg cell ablation during neonatal life causes acute T cell lymphopenia. Neonates were injected with DT at day 3 after birth, and cell suspensions from the spleen were analyzed by flow cytometry at day 3 and 4 after DT treatment (DTx). (**A–B)** Treg cell ablation efficiency in the spleens of neonates at day 4 after DTx. (**C)** Flow cytometric analysis of TCRβ^+^ T cells in the spleen of neonates at day 3 and 4 after DTx. (**D–E)** Body weight and spleen cellularity of DT- (closed squares) or control PBS-treated (open squares) neonatal *Foxp3^DTR^* mice at day 3 and day 4 after DTx. (**F)** Percentages (upper left panel) and absolute numbers (upper right panel) of TCRβ^+^ T cells, CD4^+^ T cells (lower left panel) and CD8^+^ T cells (lower right panel) in DT-treated neonatal *Foxp3^DTR^* mice. (**G)** CD44 expression in CD4^+^ (left panel) and CD8^+^ T cells (right panel), (**H**) Ki67 protein expression, and (**I**) IFN-γ production by splenic T cells from DT-treated neonatal *Foxp3^DTR^* mice; splenocytes were stimulated with PMA and Ionomycin for 4 h and cytokine production was assessed by intracellular staining; the data are shown for DT-treated and control PBS-treated mice analyzed on day 3 (closed and open circles, respectively) and day 4 (closed and open squares, respectively) after DTx. Results are representative of at least 2 independent experiments (n≥3 mice per group).

Both neonates and adult mice develop a systemic lymphoproliferative disease after Treg cell ablation. A previous study of self-reactive T cell expansion in adult *Foxp3^DTR^* mice showed a rapid increase in T cells with an activated phenotype starting at day 3–4 after diphtheria toxin (DT) injection (DTx) [12]. However, changes in the T cell compartment in neonates and in adult mice early after Treg cell ablation remain unknown. Thus, we first explored immediate changes in T cell numbers and activation state upon depletion of Treg cells in *Foxp3^DTR^* neonates at day 3 after birth, the first time point at which thymic differentiation and export of these cells to the periphery were evident [21]. As shown in Figure 1A and B, Treg cell numbers were reduced drastically by day 4 after DTx (day 7 after birth, Figure 1A). A closer examination of splenic TCRβ^+^ T cells at day 3 and 4 after DT, showed a dramatic decrease in their percentage (Figure 1C). This striking collapse in the peripheral T cell subset was not associated with DT toxicity in neonates since the body mass (Figure 1D) and the overall spleen cell counts were not significantly decreased (Figure 1E). Furthermore, 3 day-old Foxp3*^DTR/WT^* heterozygote females injected with PBS or DT did not show a reduction in the percentages of TCRβ^+^ T cells, CD4^+^ or CD8^+^ T cells, despite ablation of ∼50% of their Treg cell population on day 4 after DTx (Figure S1). Additionally, Foxp3*^DTR/WT^* heterozygote females had no appreciable weight loss compared to PBS controls.

The diminution in T cells was also reflected in percentages and absolute numbers of both CD4^+^ and CD8^+^ T cells (Figure 1F). The early onset of lymphopenia preceded massive T cell activation. Analysis of neonates on day 3 after DTx showed only slight increase in CD44 and CD69 expression, and proliferative activity as assessed by Ki67 expression in T cells compared to controls. Similarly, few T cells produced IFN-γ (Figure 1 G–I). However, by day 4 we observed a marked increase in T cell activation (Figure 1 G–I).

**Figure 2:**
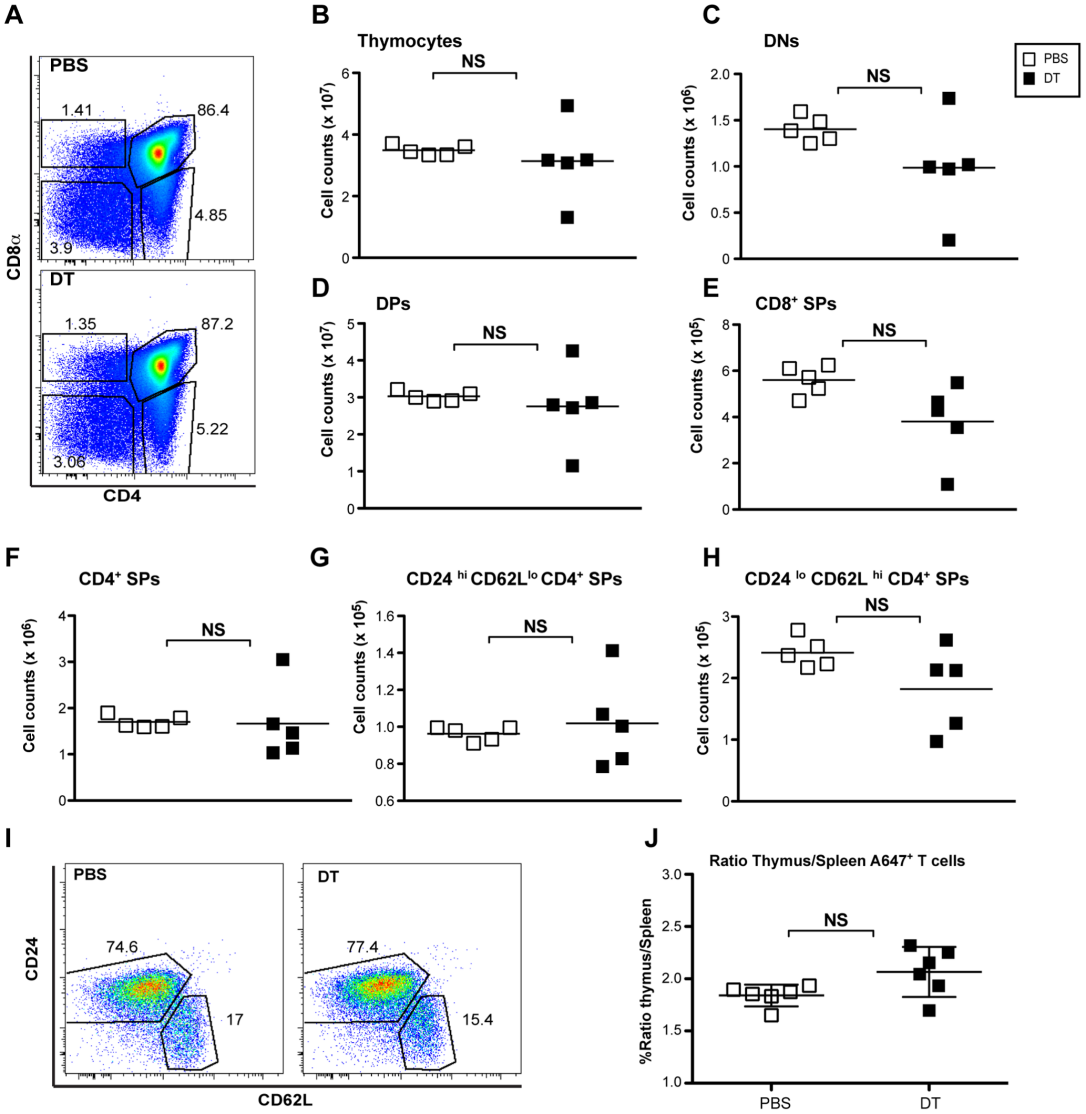
Treg cell ablation has no apparent impact on thymic function in neonates. Neonates were injected i.p with DT or PBS on day 3 after birth, and cell suspensions from the thymus were prepared on day 7 after birth. **A.** FACS analysis of CD4^+^ and CD8^+^ single positive (SP), double positive (DP) and double negative (DN) thymocytes. **B.** Comparison of thymocyte absolute numbers in DT-treated and PBS control groups. **C–E.** Absolute numbers of DNs, DPs and CD8^+^ SPs, respectively. **F–H.** Absolute numbers of CD4^+^ TCRβ^+^ SP, immature CD4^+^ CD24^hi^ CD62L^lo^ and mature CD4^+^ CD24^lo^ CD62L^hi^ SPs. **I.** Flow cytometry analysis of CD4^+^ SP subsets, as indicated in (G and H). **J.** Thymic T cell output in neonates was measured by the direct injection to the thymus of 20 µL of Alexa Fluor-647 succinimidyl ester at 10 mg/mL followed by an i.p injection of DT or PBS. Scatter plot representing the ratios of Alexa-647^+^ CD4^+^ T cells in the thymus and spleen at day 7 after birth. Results are representative of at least 2 independent experiments (n≥3 mice per group).

One possible cause for the observed lymphopenia was that thymic selection and output were sharply curtailed by Treg cell depletion. However, flow cytometric analysis showed unperturbed thymocyte subset composition (double negative (DN), double positive (DP) and single positive (SP) thymocytes) and total thymocyte counts in DT vs. control animals (Figure 2A–E). Likewise, comparable proportions of immature vs. mature (CD62L^hi^ CD24^low^) CD4^+^ SP thymocytes were found in DT treated and control neonates (Figure 2F–I). Furthermore, assessment of thymic output by *in situ* labeling of thymocytes upon injection of fluorescent dye Alexa-647 into thymic lobes of day 3 neonates followed by Treg cell ablation did not result in measurable changes in numbers of fluorescently-labeled recent thymic emigrants (Figure 2J). These results suggest that acute T cell lymphopenia after Treg cell ablation in neonates cannot be explained by perturbation of the thymic output, and exacerbated the previously reported natural lymphopenia characteristic of neonatal mice [22].

### Treg Ablation Causes Acute T Cell Lymphopenia in Adult Mice

**Figure 3:**
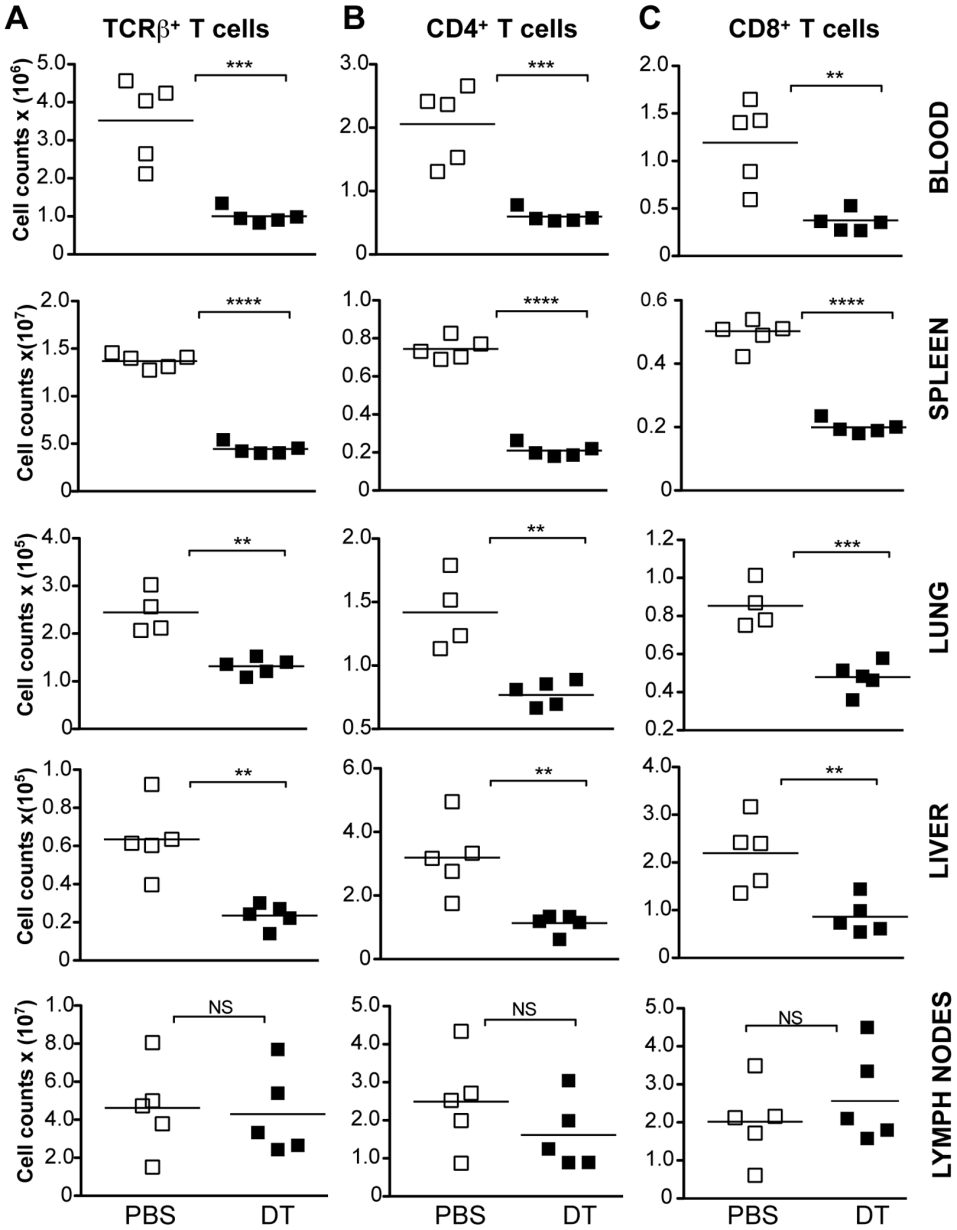
Transient acute T cell lymphopenia induced by Treg cell ablation in adult mice. Adult *Foxp3^DTR^* mice were injected i.p. with DT or PBS (control) and spleen, lymph nodes, blood, lung and livers were collected 2 days later. (**A**) Absolute numbers of TCRβ^+^ T cells (left column), (**B**) CD4^+^ T cells (middle column), and (**C**) CD8^+^ T cells (right column) in the blood, spleen, lymph nodes, lung and liver of DT- (closed squares) or control PBS-treated (open squares) *Foxp3^DTR^* mice. Results are representative of at least 2 independent experiments (n = 5 mice per group).

Next, we investigated whether adult *Foxp3^DTR^* mice were also prone to develop T cell lymphopenia upon Treg cell ablation. In these mice, we also observed a generalized acute drop in CD4^+^ and CD8^+^ T cell numbers (Figure 3A, left panel; Figure 3B, middle panel; Figure 3C, right panel) in the blood, spleen, lung, and liver. Lymph nodes were the only site, where a pronounced decrease in T cell numbers was not observed (Figure 3). These data indicate that lymphopenia is a common feature associated with Treg cell ablation regardless of the age of animals.

**Figure 4:**
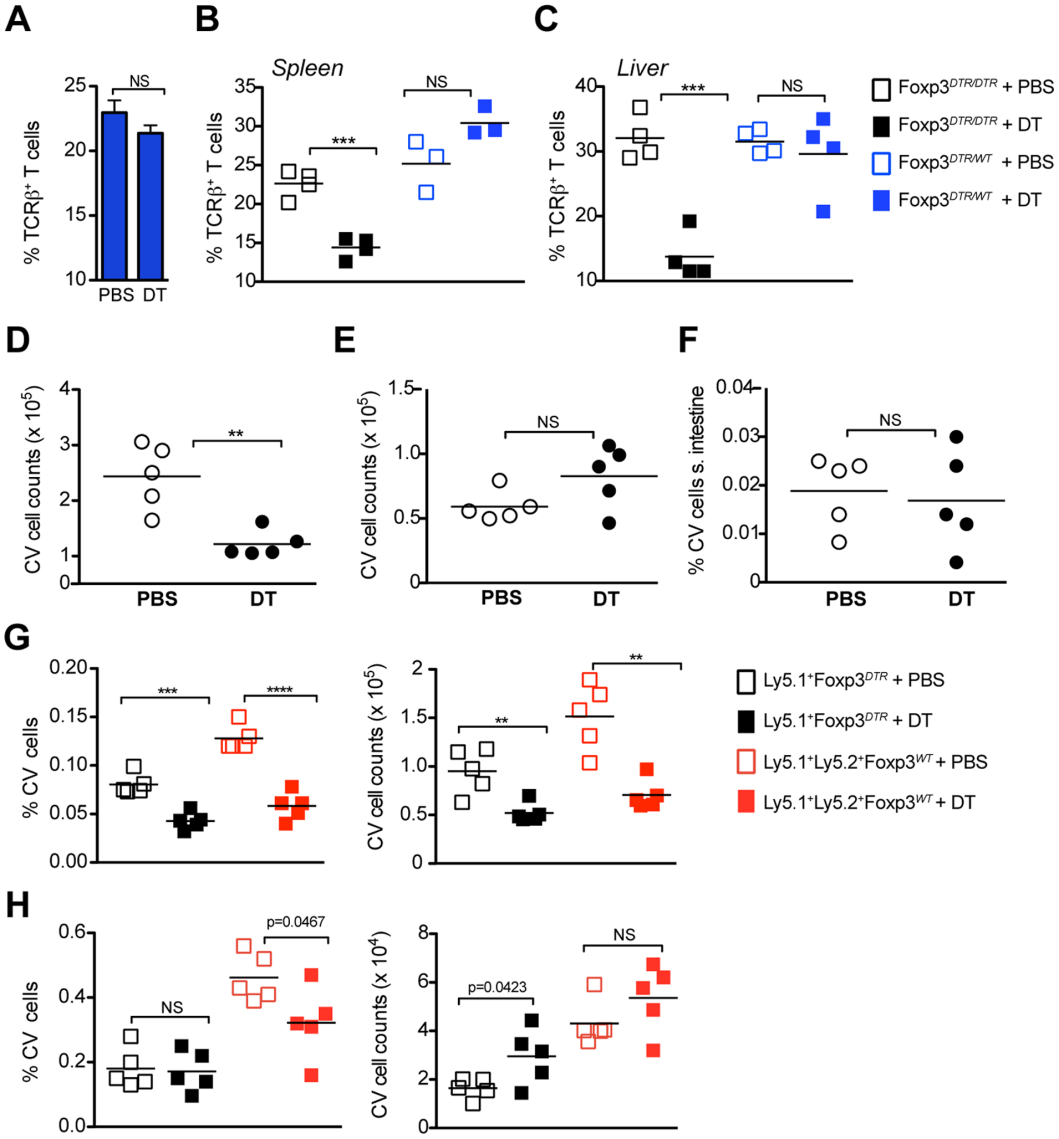
T cell lymphopenia is not triggered by non-specific DT toxicity, leaky DTR expression, or T cell redistribution. (**A**) WT mice were injected i.p. with DT and splenic T cell subsets were analyzed by flow cytometry two days later. Bar graph represents the percentage of TCRβ^+^ T cells in the spleen. (**B–C**) Homozygote *Foxp3^DTR/DTR^* and heterozygote *Foxp3^DTR/WT^* female mice were injected i.p with DT or PBS (control); spleen and liver T cell subsets were analyzed two days later by flow cytometry. Percentages of TCRβ^+^ T cells in the spleen (**B)** and liver (**C**) in DT-treated *Foxp3^DTR/DTR^* (closed black squares) or *Foxp3^DTR/WT^* (closed blue squares) and in PBS-treated *Foxp3^DTR/DTR^* (open black squares) or *Foxp3^DTR/WT^* (open blue squares). (**D–G**) CellTrace Violet-labeled T cells (CV T cells) from Ly5.1 *Foxp3^DTR^* mice and Ly5.1/Ly5.2 *Foxp3^WT^* mice were adoptively transferred into Ly5.2 *Foxp3^DTR^* mice and 24 hours later the recipients were injected with DT or PBS. Donor and host T cell subsets were analyzed by flow cytometry 2 days after DTx. Total numbers of Ly5.1^+^ and Ly5.1/Ly5.2^+^ CV T cells in (**D**) the spleen, (**E**) lymph nodes, and (**F**) small intestine. Percentages and absolute numbers of Ly5.1^+^ and Ly5.1/Ly5.2^+^ CV T cells in the spleen (G.) and lymph nodes (H.) on day 2 post-treatment in DT treated (Ly5.1/Ly5.2^+^ *Foxp3^WT^* CV T cells - closed black squares; Ly5.1^+^ *Foxp3^DTR^* CV T cells – closed red squares) or control PBS-treated mice (Ly5.1/Ly5.2^+^ *Foxp3^WT^* CV T cells - open black squares; Ly5.1^+^ *Foxp3^DTR^* CV T cells – open red squares). Results are representative of at least 2 independent experiments (n = 5 mice per group).

To exclude the possibility that in adults, unlike neonates, an early decline in T cell numbers caused by the Treg cell depletion was due to sizable T cell redistribution to other locations, we transferred CellTrace Violet (CV)-labeled naive Ly-5.1^+^ and Ly5.1^+^Ly5.2^+^ T cells isolated from *Foxp3^DTR^* and *Foxp3^WT^* mice, respectively, into Ly5.2^+^ *Foxp3^DTR^* recipients a day prior to DTx. Both host and adoptively transferred CV-labeled T cells showed a similar marked reduction in the spleen, but not in the lymph nodes upon DTx (Figure 4D–E; data not shown). Additionally, we did not observe an enrichment of T cells in the small intestine (SI) (Figure 4F) that serves as a major disposal route for T cells undergoing activation-induced cell death [23]. Thus, diminished T cell numbers in the periphery of mice subjected to an acute Treg cell ablation were not caused by the disposal of T cells in the gut.

These experiments also excluded transient expression of DTR in non-Treg cells after Treg cell depletion as another trivial explanation for the observed phenomenon. Adoptively transferred CV-labeled naïve *Foxp3^DTR^* and *Foxp3^WT^* T cells showed a comparable reduction in the spleen (Figure 4G–H). Thus, CV-labeled T cells from *Foxp3^DTR^* mice were not selectively susceptible to DTx. Similarly to experiments performed in neonates, DT treatment of *Foxp3^DTR/WT^* mice did not reveal a decline in T cell numbers in lymphoid and non-lymphoid organs as compared to *Foxp3^WT^* mice (Figure 4A–C). If transient DTR expression in non-Treg cells were to account for the observed decline in their numbers in DT-treated *Foxp3^DTR^* mice, the T cell numbers in DT-treated *Foxp3^DTR/WT^* should have declined by 50% of the observed decrease in DT-treated *Foxp3^DTR/DTR^* females.

In aggregate, these results suggest that diminished T cell numbers observed upon acute Treg cell ablation represented lymphopenia and cannot be explained by non-specific toxicity associated with DT or leaky expression of DTR in Foxp3^−^ “non-Treg” cells, T cell redistribution to non-lymphoid organs or their disposal in the gut.

### T Cell Lymphopenia is not Mediated by Apoptotic-cell Death and is not Corrected by Homeostatic Growth Factors

**Figure 5:**
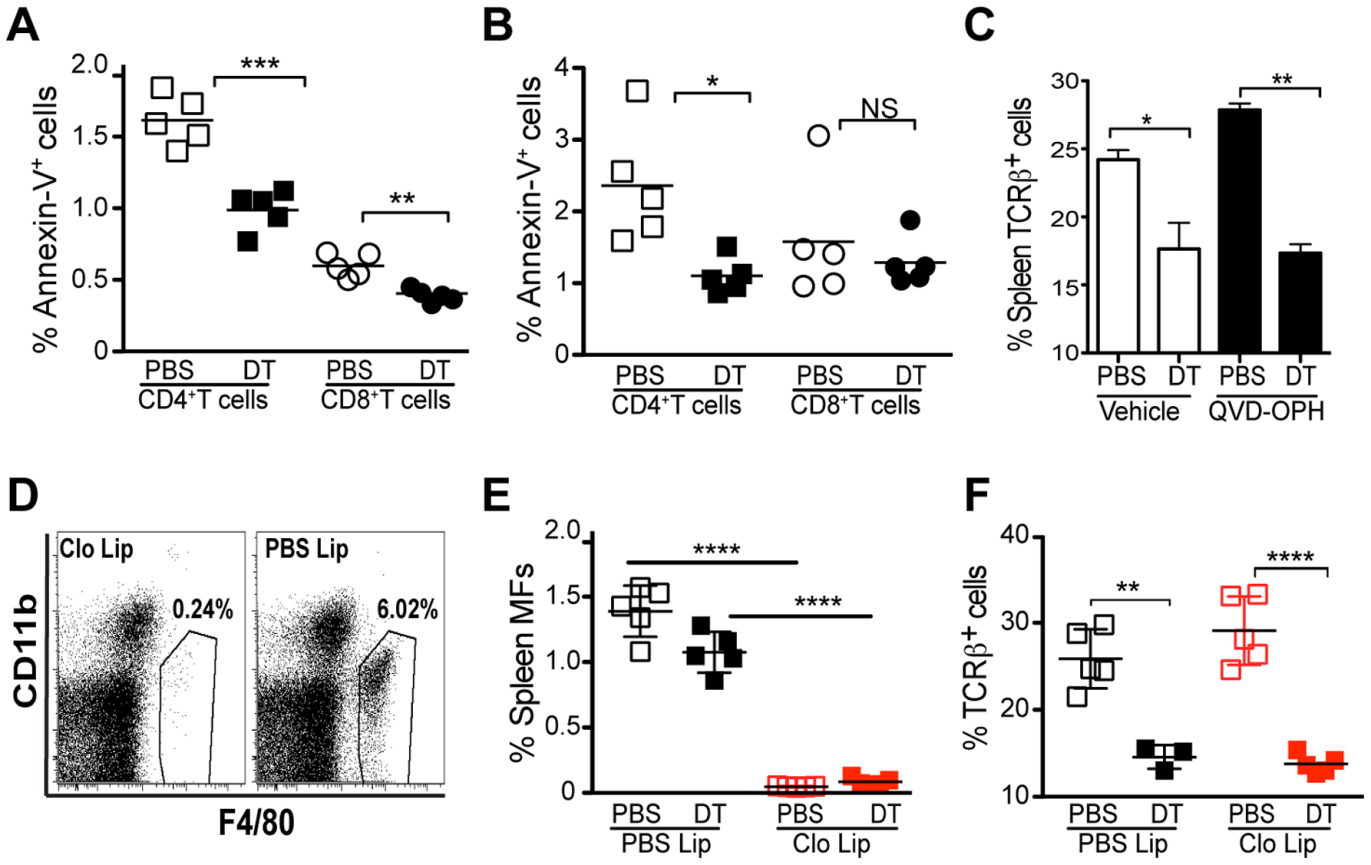
Apoptotic T cell death or MF engulfment do not account for Treg cell depletion mediated transient lymphopenia. Spleen and lymph node cells from DT- or PBS-treated *Foxp3^DTR^* mice were stained with Annexin-V and analyzed by flow cytometry. Percentage of Annexin-V^+^ CD4^+^ (**A**) and CD8^+^ T cells (**B**) in DT- (closed circles) or PBS-treated (open circles) *Foxp3^DTR^* mice. (**C**) Caspase inhibitor QVD-OPH was administered i.p. into *Foxp3^DTR^* mice on the day of, and on day 1 after DT injection. Percentages of splenic TCRβ^+^ T cells in QVD-OPH treated or control mice on day 2 after DT or PBS treatment. Clodronate encapsulated (Clo-lip) or control (PBS-lip) liposomes were injected i.v. into *Foxp3^DTR^* mice 10 days prior to DTx. 2 days after DT injection, splenic T cells were analyzed by flow cytometry. (**D**) Flow cytometric analysis of red pulp MF depletion in Clo-lip treated mice. Percentage of red pulp MF depletion (**E**) and spleen TCRβ^+^ T cells (**F**) in DT treated *Foxp3^DTR^* mice injected with Clo-lip (closed red squares) or PBS-lip (closed black squares) and in PBS treated *Foxp3^DTR^* mice injected with Clo-lip (open red squares) or PBS-lip (open black squares). Results are representative of at least 2 independent experiments (n≥3 per group).

T cell lymphopenia may be caused by different mechanisms including apoptotic T cell death. We evaluated T cell apoptosis by Annexin-V staining at day 2 after DTx. The percentage of apoptotic T cells did not increase but rather decreased compared to control mice (Figure 5A–B). Similarly, a detailed kinetic examination showed a steady decline in apoptotic cell percentage from the time of DTx to day 2 (data not shown). Further, the *in vivo* administration of a potent cell-permeable apoptotic inhibitor, QVD-OPH [24], did not prevent T cell lymphopenia after Treg cell ablation (Figure 5C). These results suggest that apoptotic cell death does not play a role in this phenomenon.

IL-7 is an essential cytokine required for T cell survival in secondary lymphoid organs [25]. If a decrease in IL-7 expression was responsible for T cell lymphopenia, exogenous IL-7 administration should reverse it. Despite an observed decrease in IL-7 and CCL21 transcripts in the lymph nodes and spleen of DT treated mice (Figure S2), administration of IL-7 failed to rescue T cell lymphopenia (Figure S2). These results argue that the observed T lymphopenia was not due to down-modulation of IL-7 caused by Treg cell depletion.

### Lymphopenia is not Mediated by Macrophage-mediated Engulfment of T Cells

Dendritic cells (DCs) and macrophages (MFs) have been implicated in T cell peripheral tolerance due to their ability to promote Treg cell induction [26], [27] and deletion of self-reactive T cells [28]. Furthermore, Treg cells target APCs such as DCs by limiting surface costimulatory molecule and MHC expression necessary for T cell priming [29]. MF mediated clearance of T cells has also been implicated as a mechanism of attenuation of graft versus host disease [30]. Since we observed rapid onset of T cell lymphopenia in the absence of a detectable increase in apoptosis, we tested if clearance of T cells by MFs could account for the observed lymphopenia upon Treg cell depletion. We combined Treg cell ablation and MF depletion mediated by clodronate-loaded liposomes. Pronounced depletion of red-pulp MFs was observed on day 10 after i.v clodronate liposome injection and was maintained after DTx (Figure 5D and E). However, MF depletion in *Foxp3^DTR^* mice did not prevent development of T cell lymphopenia (Figure 5F). Together, these experiments suggested that lymphopenia elicited early after Treg cell depletion was unlikely caused by MF-mediated removal of T cells.

Our study revealed that Treg cell depletion results in an acute T cell lymphopenia preceding self-reactive T cell expansion in both adult and neonatal mice. Treg cells prevented a loss of peripheral T cells in the spleen, blood, and non-lymphoid tissues, but not in the lymph nodes. This lymphopenia commenced immediately after Treg cell depletion in both lymphoreplete adult and naturally lymphopenic neonatal mice and was transient since T cell numbers recovered by day 6 after DTx in adult mice (data not shown). Although the mechanism of the observed lymphopenia caused by the Treg elimination remains unknown, we excluded non-specific DT toxicity or leaky DTR expression in Foxp3^−^ “non-Treg” cells, T cell apoptosis, T cell redistribution to non-lymphoid tissues, and withdrawal of the essential growth factor, IL-7. Additionally, thymic output and thymocyte subset composition were not altered coincidentally with the observed lymphopenia.

T cell sequestration in lymphoid organs affecting simultaneously the spleen and lymph nodes has been described during acute responses to superantigens and LPS [31]. However, this phenomenon is unlikely to explain our results since tissues were collagenase digested and dissociated with buffers containing EDTA and routinely, cell suspensions obtained from lymphoid organs without collagenase treatment showed similar T cell numbers (data not depicted). On the other hand, the early loss of T cells either in neonates or adult mice was largely limited to the naïve T cell compartment. Adoptive transfer of naïve CV-T cells supported this observation, and a close examination of the remaining T cells showed negligible signs of T cell activation, as shown for CD44 and CD62L expression (data not shown).

Since T cell numbers decline very rapidly after Treg cell elimination, peripheral deletional tolerance caused by DCs or other APC types is unlikely to explain our findings because it requires cell division spanning several days before T cells die from apoptosis [28], [32]. Although rapid clearance of live T cells by MFs has been reported in particular settings [30], we failed to find evidence in support of this mechanism at least in the spleen. In addition to MFs, natural killer cells can attack T cells [33]. However, depletion of these cells did not alleviate T cell lymphopenia (data not shown). Nonetheless, it remains possible that cells of hematopoietic or non-hematopoietic origin other than macrophages may clear T cells by an alternative mechanism triggered by Treg cell elimination resulting in the acute T cell lymphopenia in the spleen and non-lymphoid tissues. In this regard, recent studies have shown the role of non-hematopoietic cells such as hepatocytes in the clearance of activated T cells to avoid tissue destruction and enforce peripheral tolerance [34].

Regardless of a yet to be defined mechanism, our observations raise a question as to whether transient lymphopenia early after Treg cell depletion contributes to the subsequent massive T cell activation. Whether TCR affinity and specificity for “self” antigens are critical for this attrition of T cells remains to be determined.

## Supporting Information

Figure S1 **Neonatal T cell lymphopenia is not associated with unspecific DT toxicity.** Neonate Foxp3*^DTR/WT^* heterozygote female mice were injected i.p either with DT or PBS at day 3 after birth. 4 days after DTx neonates were weighted and the spleen was isolated and T cells were analyzed by flow cytometry. Flow cytometric analysis showing the percentage of TCRβ^+^ T cells (**A**) CD4^+^ and CD8^+^ T cells (**B**) and the Treg cell ablation efficiency (**C**) in the spleen of neonate Foxp3*^DTR/WT^* female mice injected with DT or PBS. (**D**) Bar graph representation of the percentage of TCRβ+ T cells in Foxp3*^DTR/WT^* heterozygote neonate female mice treated with PBS or DT at day 4 after DTx. (**E**) Foxp3*^DTR/WT^* neonate body weight at day 4 after DTx (n≥3 mice per group).
(PDF)

Figure S2 **qPCR analysis of IL-7 and CCR7 ligands transcripts in the spleen and lymph nodes of DT treated mice.** qPCR analysis of IL-7 (**A**), CCL21(**B**) and CCL19 (**C**) transcripts in the spleen (right column) and lymph nodes (left column) from mice treated with DT at different time points. (**D**) *Foxp3^DTR^* mice were injected with DT or PBS and treated or mock treated with rh-IL7 and at day 2 after DT injection, spleens and cell suspensions were prepared for flow cytometry. Scatter plot representing the percentages of TCRβ^+^ T cells in the spleen from the different experimental groups. Open circles (PBS control group), closed circles (DT group), open squares (PBS control group+rh-IL7), and closed squares (DT group+rh-IL7).
(PDF)
