# Correction: Regulatory T Cell Ablation Causes Acute T Cell Lymphopenia

**DOI:** 10.1371/journal.pone.0099340

**Published:** 2014-05-20

**Authors:** The PLOS ONE Staff

In the Methods section, the authors found a mistake regarding the dose of diphtheria toxin (DT) used to challenge the mice described in the methods section:

"*In vivo* Treg Cell Ablation

Treg cell ablation in adult *Foxp^3DTR^* mice was accomplished by a single i.p injection of DT (Sigma) at a dose of 50 mg/kg…"

50 mg/kg should be corrected to 50 µg/kg.
