# Supplementary material for: Regulatory T Cell Ablation Causes Acute T Cell Lymphopenia
Source: PLoS One. 2014 Jan 23;9(1):e86762. doi: 10.1371/journal.pone.0086762 (PMC3900634; doi:10.1371/journal.pone.0086762)
Supplement: Figure S1 — Neonatal T cell lymphopenia is not associated with unspecific DT toxicity. Neonate Foxp3DTR/WT heterozygote female mice were injected i.p either with DT or PBS at day 3 after birth. 4 days after DTx neonates were weighted and the spleen was isolated and T cells were analyzed by flow cytometry. Flow cytometric analysis showing the percentage of TCRβ+ T cells (A) CD4+ and CD8+ T cells (B) and the Treg cell ablation efficiency (C) in the spleen of neonate Foxp3DTR/WT female mice injected with DT or PBS. (D) Bar graph representation of the percentage of TCRβ+ T cells in Foxp3DTR/WT heterozygote neonate female mice treated with PBS or DT at day 4 after DTx. (E) Foxp3DTR/WT neonate body weight at day 4 after DTx (n≥3 mice per group). (PDF) [file pone.0086762.s001.pdf]

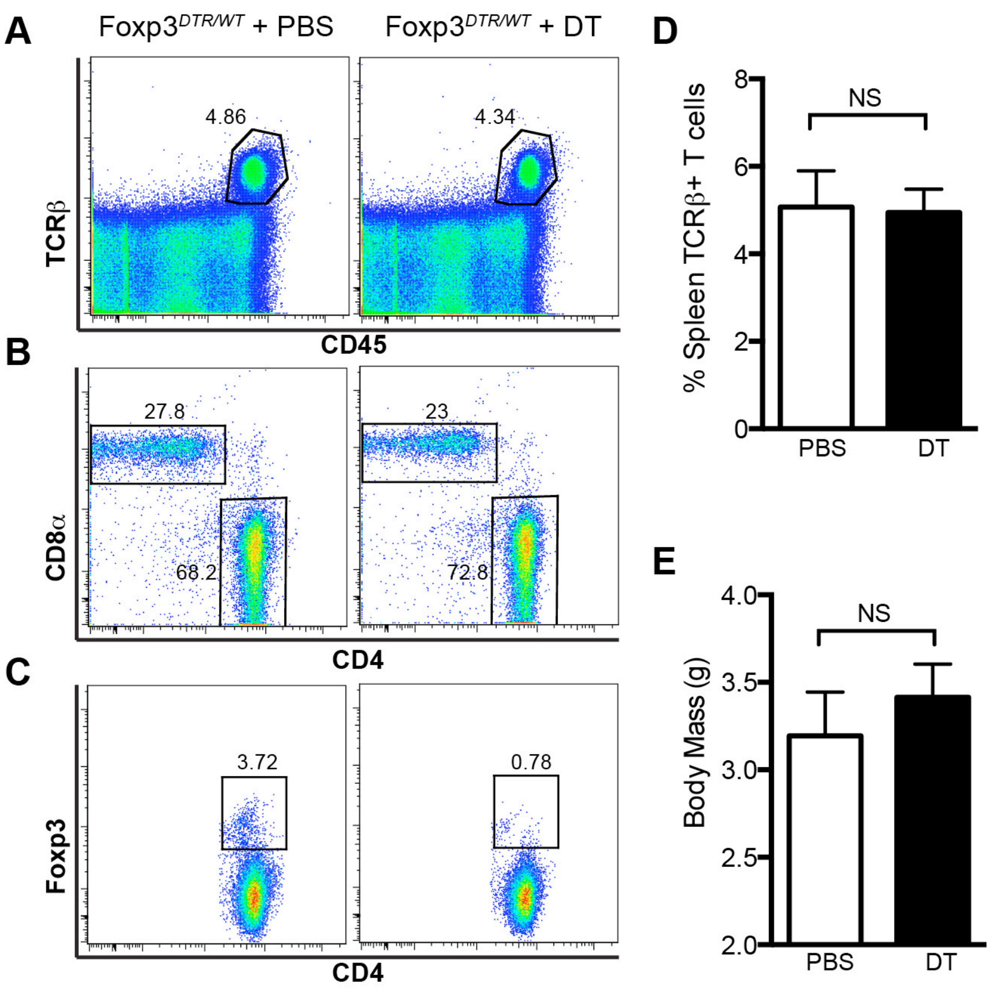

Figure S1. Neonatal T cell lymphopenia is not associated with unspecific DT toxicity. Neonate Foxp3 $^{DTR/WT}$ heterozygote female mice were injected i.p either with DT or PBS at day 3 after birth. 4 days after DTx neonates were weighted and the spleen was isolated and T cells were analyzed by flow cytometry. Flow cytometric analysis showing the percentage of TCRβ^{+} T cells (**A**) CD4^{+} and CD8^{+} T cells (**B**) and the Treg cell ablation efficiency (**C**) in the spleen of neonate Foxp3$^{DTR/WT}$ female mice injected with DT or PBS. (**D**) Bar graph representation of the percentage of TCRβ^{+} T cells in Foxp3$^{DTR/WT}$ heterozygote neonate female mice treated with PBS or DT at day 4 after DTx. (**E**) Foxp3$^{DTR/WT}$ neonate body weight at day 4 after DTx (n≥3 mice per group).
