# Supplementary material for: Regulatory T Cell Ablation Causes Acute T Cell Lymphopenia
Source: PLoS One. 2014 Jan 23;9(1):e86762. doi: 10.1371/journal.pone.0086762 (PMC3900634; doi:10.1371/journal.pone.0086762)
Supplement: Figure S2 — qPCR analysis of IL-7 and CCR7 ligands transcripts in the spleen and lymph nodes of DT treated mice. qPCR analysis of IL-7 (A), CCL21(B) and CCL19 (C) transcripts in the spleen (right column) and lymph nodes (left column) from mice treated with DT at different time points. (D) Foxp3DTR mice were injected with DT or PBS and treated or mock treated with rh-IL7 and at day 2 after DT injection, spleens and cell suspensions were prepared for flow cytometry. Scatter plot representing the percentages of TCRβ+ T cells in the spleen from the different experimental groups. Open circles (PBS control group), closed circles (DT group), open squares (PBS control group+rh-IL7), and closed squares (DT group+rh-IL7). (PDF) [file pone.0086762.s002.pdf]

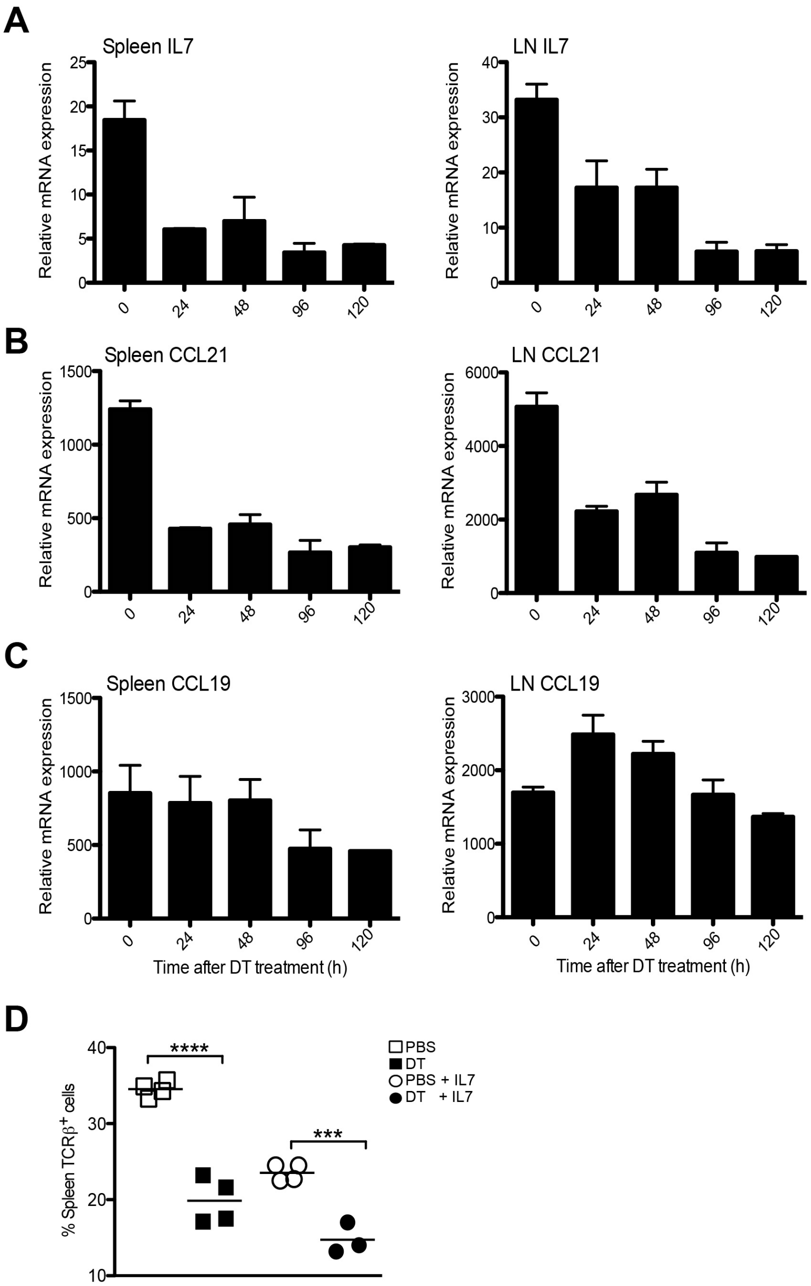

Figure S2. **qPCR analysis of IL-7 and CCR7 ligands transcripts in the spleen and lymph nodes of DT treated mice.** qPCR analysis of IL-7 (**A**), CCL21(**B**) and CCL19 (**C**) transcripts in the spleen (right column) and lymph nodes (left column) from mice treated with DT at different time points. (**D**) *Foxp3*^{DTR} mice were injected with DT or PBS and treated or mock treated with rh-IL7 and at day 2 after DT injection, spleens and cell suspensions were prepared for flow cytometry. Scatter plot representing the percentages of TCRβ^{+} T cells in the spleen from the different experimental groups. Open circles (PBS control group), closed circles (DT group), open squares (PBS control group + rh-IL7), and closed squares (DT group + rh-IL7).
